# Resilience and Depressive Symptoms among Medical Staff in a Military Hospital Dedicated to the Treatment of COVID-19

**DOI:** 10.3390/ijerph191811576

**Published:** 2022-09-14

**Authors:** Chorom Lee, Byungyoon Yun, Won-Tae Lee, Juho Sim, Chi-Nyon Kim, Jong-Uk Won, Jin-Ha Yoon

**Affiliations:** 1 Department of Preventive Medicine, Yonsei University College of Medicine, Seoul 03722, Korea; 2 Department of Occupational and Environmental Medicine, Severance Hospital, College of Medicine, Yonsei University, Seoul 03722, Korea; 3 Department of Public Health, Yonsei University Graduate School, Seoul 03722, Korea; 4 The Institute for Occupational Health, Yonsei University College of Medicine, Seoul 03722, Korea

**Keywords:** resilience, depression, COVID-19, military hospital, medical staff, mental health

## Abstract

Coronavirus disease 2019 (COVID-19) is prevalent around the world, and many studies suggest that depression among medical staff is on the rise during the pandemic. This study aims to assess the relationship between depressive symptoms and individual resilience among military hospital personnel responsible for treating patients with COVID-19. Individuals from the Armed Forces Daejeon Hospital who responded to the questionnaires from 8 February to 15 February 2022 participated in this study. Resilience and depressive symptoms were measured via the Korean Resilience Quotient Test-53 and Patient Health Questionnaire-9, respectively. We employed multivariable logistic regression analysis to estimate Odds Ratios (ORs) and 95% Confidence Intervals (CIs) of depressive symptoms. Among 181 participants, the individuals with depressive symptoms and high resilience accounted for 8.8% and 61.9%, respectively. Significant correlations between depressive symptoms and both the low resilience and low resilience positivity groups were found (adjusted OR 10.30 [95% CI 1.74–61.01] and OR 13.90 [95% CI 1.93–100.02], respectively). This study notes a significant inverse relationship between depressive symptoms and resilience even after adjusting for demographic and occupational characteristics. To overcome depressive symptoms among hospital personnel, it is necessary to seek ways to improve individual resilience, especially positivity.

## 1. Introduction

The unprecedented emergence of COVID-19, with an exponential increase in the confirmed cases and fatalities, has resulted in a significantly increased burden of mental disorders, including depressive and anxiety disorders [1,2,3]. Numerous studies have sought to determine the short- and long-term effects of a traumatic pandemic on the mental health of a variety of populations [4,5,6,7,8]. Medical staff who came in close contact with suspected or confirmed COVID-19 patients experienced physical and psychological pressure owing to the risk of exposure to infectious diseases as well as overwork, during the pandemic period [8,9,10]. About one-fifth of the medical staff attending to COVID-19 patients in hospitals in China experienced depression [11]. Compared to mid-Eastern respiratory syndrome (MERS) or seasonal influenza, anxiety among healthcare personnel dealing with COVID-19 was noticeably higher. The most frequent worry was spreading the disease to friends and family rather than being infected themselves [12].

Compared with non-frontline medical professionals, frontline medical staff reported higher levels of occupational stress and danger [13,14]. Additionally, there was a positive correlation between the signs of mental health and professional stress. During the COVID-19 outbreak, there was a correlation between work stress and mental health symptoms in both frontlines and support staff medical personnel [15]. Moreover, active-duty personnel suffered from more physical symptoms and emotional stress than civilian employees [16,17]. Regarding the special environment of military hospitals, those who have to rapidly and flexibly operate their missions related to the widespread infectious disease as well as their original tasks may experience more serious psychological and physical stress, which could result in various mental health concerns.

Some individuals occasionally experienced depression and other psychiatric problems owing to acute or chronic stress [18]. Others, however, learned resourcefulness and growth, resulting in self-righting and psychological performance which was significantly higher than the expectations based on one’s abilities and experiences [19]. Several studies have explained this difference using the term “resilience,” which refers to the positive power to overcome trials and hardships through learning and training, to finally return to one’s original state [20,21,22,23]. Resilient individuals tend to exhibit fewer depressive symptoms and are at lower risk of developing persistent depression [24,25]. Given the same dangers, individual differences in the ability to adapt to trauma and stress are accounted for by viewing resilience as a trait [26]. However, studies on the relationship between the depressive symptoms and resilience of medical staff regarding COVID-19 are lacking. Particularly, no study has examined the association between psychological health and resilience among military hospital personnel.

Therefore, the hypothesis of whether resilience and its subtypes are closely related to the depressive symptoms of medical staff should be investigated. This study aims to assess the relationship between depressive symptoms and individual resilience among military hospital personnel responsible for treating patients with COVID-19 and provide basic data for establishing mental health management guidelines.

## 2. Materials and Methods

### 2.1. Study Design

This study was conducted among personnel from the Armed Forces Daejeon Hospital in Korea who had attended to suspected and confirmed COVID-19 patients for at least one year from December 2019 to December 2021. The questionnaire was distributed to 250 people who understood the purpose of the study; 185 participants voluntarily participated in this study from 8 February to 15 February 2022. A total of 181 participants were included in the final analysis, after the content of the questionnaire was reviewed, then four participants having missing values on the questionnaire were excluded (Figure S1). Before data collection, the contents of the questionnaire were reviewed for security and approved by Armed Forces Daejeon Hospital in Korea. To minimize contact owing to COVID-19, the purpose of the study and survey cooperation were explained via the internet. A link to an electronic questionnaire and a QR code were further distributed so that the participants could directly indicate their willingness to participate in the survey. 

This study was approved by the Clinical Research and Protection Center (IRB) of the Armed Forces Medical Command (IRB: AFMC-202201-HR-004-01). Prior to data collection, electronic written informed consent was obtained from each participant.

### 2.2. Measurements

#### 2.2.1. Demographic and Occupational Data (Table 1)

Gender, age, and marital status were investigated as demographic variables of the study participants. Age was classified into 20s, 30s, and 40s or more. Marital status was classified as married or single. Occupational variables included occupation, direct work experience with COVID-19 management, work department (only for those with experience in COVID-19 management), total clinical career, and past medical support experience for MERS. Occupations were classified into doctors, nurses, nursing assistants, medical technicians, and administrative personnel. Administrative staff included the administration department, human resources administration department, and logistics department, which are in charge of administrative support for responding to COVID-19 in the hospital, and security personnel responsible for entering and guiding COVID-19 patients in and out of the hospital. 

Regarding work experience, participants who responded “yes” to the question “experience of direct work experience in the treatment and nursing of COVID-19 patients” were classified into the direct work experience group, while the others were classified as the indirect work experience group. The types of work were stratified into isolation units and screening clinics. The total clinical career was classified as less than one year, 1–3 years, 4–6 years, 7–9 years, and 10 years or more, including private clinical careers. Medical support for MERS was classified as “yes” or “no” according to past experience with medical support in the event of MERS.

#### 2.2.2. Korean Resilience Quotient Test-53 (KRQ-53)

Reivich and Shatte first developed an index to evaluate resilience [27]. Kim Joo-hwan modified this index to the KRQ-53 to reflect the situation in Korea [21,22]. This test, comprising 53 items, is divided into three sub-categories—self-regulation, connections, and positivity. Self-regulation contains 18 items that evaluate emotion control, impulse control, and cause-analysis abilities. Connections includes 18 items that evaluate communication, empathy, and self-expanding abilities. Positivity comprises 17 items that evaluate self-optimism, life satisfaction, and gratitude. Each item is rated on a 5-point Likert scale ranging from “not at all” (1 point) to “strongly agree” (5 points). A higher score indicates a higher level of resilience. The 24 inverse questions were calculated by converting them into inverse scoring questions; the total score ranges from 53 to 265 points [22]. Participants obtaining more than 195 out of a total of 265 points were categorized as the high-resilience group, while others formed the low-resilience group [21,28].

#### 2.2.3. Patient Health Questionnaire-9 (PHQ-9)

We adopted the PHQ-9 to evaluate depressive symptoms. The PHQ-9 is a self-report questionnaire designed to detect primary mental disorders and assist in the diagnosis of major depressive disorder. It is an evaluation tool composed of nine items that correspond to the diagnostic criteria of the Diagnostic and Statistical Manual of Mental Disorders. Owing to the small number of questions and the relatively short test time, it is used to quickly screen for depressive disorders in primary care, clinical settings, and questionnaires. The PHQ-9 is a reliable and valid tool for evaluating the severity of depressive symptoms. Scores are calculated by summing up the items on how often depressive symptom-related problems have been experienced in the past two weeks. The higher the PHQ-9 score, the higher the severity of depressive symptoms [29]. Regarding depressive symptoms, 10 out of a total of 27 points were set as the cut-off point. Variables were created based on the view that a score of 10 or more represented a group experiencing depressive symptoms, and a score of 0–9 points represented a group not experiencing depressive symptoms [11,30].

### 2.3. Statistical Analysis

The chi-square test was performed for categorical variables. A t-test was further performed for continuous variables. A chi-square test was performed to identify the distribution of depressive symptoms and differences in groups on resilience and the sub-categories of resilience. A multivariable logistic regression model was used to estimate the odds ratio (OR) and 95% confidence interval (CI), and to confirm the relationship between depressive symptoms and resilience and its sub-categories (self-regulation, connections, and positivity) among the sample. We adjusted for demographic characteristics (age, gender, and marital status) and occupational characteristics (occupation, medical experience for suspected or confirmed COVID-19 cases, total clinical career, and past medical support experience). Models 2 and 3 included adjustments for demographic characteristics and occupational characteristics as well as demographic characteristics, respectively. However, Model 1 did not include any adjustment. Statistical significance was set at *p* < 0.05. All statistical analyses were performed using R cran 4.2.

## 3. Results

There are 120 (66.3%) females and 61 (33.7%) males in the current analysis. The participant numbers of each age group are 36 (19.9%), 79 (43.6%), and 66 (36.5%) in the 20–29, 30–39, 40 or more age groups, respectively. Of all study subjects, 89 (49.2%) were married and 92 (50.8%) were single. The participant number of each occupation group are 24 (13.3%), 71 (39.2%), 47 (26.0%), 18 (9.9%), and 21 (11.6%) in doctor, nurse, nurse’s aide, medical engineer, and administrative staff occupation group, respectively. There are 133 (73.5%) group with medical experience for suspected or confirmed COVID-19. Of these, 106 (79.7%) worked in isolation unit and 27 (20.3%) worked in screening clinic. The participant number of each total clinical career group are 29 (16.0%), 22 (12.2%), 60 (33.10%), 38 (21.0%), and 32 (17.7%) in less than 1 year, 1–3, 4–6, 7–9, more than 10 years total clinical career group, respectively. The group with medical support experience was 57 (31.5%). There are different distribution of age group between genders. The highest proportion of age group is 40 or more in female (58 with 48%), and 30-39 in male (40, 66%) (*p* < 0.001). The proportion marital status was not differing across genders (Table S1).

Among the 181 participants, 8.8% exhibited depressive symptoms; 61.9% showed high resilience. The prevalence of depressive symptoms and resilience differed insignificantly according to gender, age, marital status, occupational group, contact with COVID-19 patients, or total clinical career cases (all *p* > 0.05, Table 1 and Table 2). Depressive symptoms in the high- and low-resilience groups were 1.8% (n = 2) and 20.3% (n = 14), respectively, showing a significant statistical difference (*p* < 0.001). In the group with low self-regulation of resilience (19.0%, *p* = 0.001), the group with low resilience positivity (18.1%, *p* < 0.001) had a higher rate of depressive symptoms than the group with high resilience (Table 1).

According to the logistic regression models, both the low resilience and low resilience positivity groups showed significant associations with depressive symptoms (adjusted OR 10.30 [95% CI 1.74–61.01] and OR 13.90 [95% CI 1.93–100.02], respectively), while self-regulation and connection were insignificantly related with depressive symptoms in Model 3 (adjusted OR, 2.45 [95% CI, 0.55–10.87] and OR 1.69 [95% CI 0.38–7.39], respectively) (Table 3). 

In Model 1, the groups with low resilience, low self-regulation of resilience, and low resilience positivity showed a significantly higher prevalence of depressive symptoms than the group with high resilience (crude OR [95% CI] of 2.00 [1.45–2.76], 2.51 [1.79–3.52], and 1.80 [1.31–2.47], respectively). In Model 2, the groups with low resilience, low self-regulation of resilience, and low resilience positivity showed higher experiences of depressive symptoms than the group with high resilience (adjusted OR [95% CI] of 8.87 [3.30–23.84], 2.33 [1.06–5.11], and 2.45 [1.14–5.28], respectively). In conclusion, in Model 3 with the group with low resilience, the experience of depressive symptoms was higher in the group with low resilience positivity than in the group with high resilience (adjusted OR [95% CI] of 10.30 [1.74–61.01] and 13.90 [1.93–100.02], respectively).

## 4. Discussion

This study notes a significant inverse relationship between depressive symptoms and resilience even after adjusting for demographic and occupational characteristics. According to the analysis related to the sub-categories of resilience, positivity was significantly related with none of the depressive symptoms. 

We found that the individuals with depressive symptoms and high resilience accounted for 8.8% and 61.9% of the sample, respectively. This study is similar to those conducted among nurses working in respiratory clinical areas, in COVID-19 management hospitals; the groups that exhibited depressive symptoms and moderate or high resilience accounted for 17.2% and 65% of the sample, respectively [31]. During the COVID-19 outbreak, the estimated self-reported rate of anxiety and depressive symptoms among medical staff was higher than that of the general population [32]. During the pandemic, the workload of hospital staff significantly increased. Consequently, they experienced a lot of work-related stress, burnout, depression, and anxiety [33]. Therefore, medical staff must have access to adequate mental health support. 

Previous studies show that resilience alleviates the development of depression [34,35]. Enhancing stress resilience is decreasing the likelihood of developing stress-induced depression/anxiety, and treating stress-induced psychopathology with potential psychological, social, spiritual, and neurobiological approaches [18]. Particularly, resilience and emotional regulation play an important role in reducing depression, anxiety, and stress among hospital staff during the COVID-19 pandemic [36].

Resilience is a dynamic concept that includes positive adaptation and personal development in changing and challenging environments [37]. The development of positive emotional granularity may be a mechanism by which resilient individuals acquire superior coping skills; this knowledge allows them to adapt flexibly and voluntarily to negative situations [38]. According to previous research, positive emotions can improve the ability to handle stress [39]. Improved coping consequently reinforces resilience [38]. Particularly, the study’s results show that the quality of the positive emotions experienced by healthcare workers needs to be improved to increase their psychological resilience while working during the COVID-19 pandemic [40]. A previous review suggested the presence of an inverse relationship between mental disorders such as depression, anxiety, somatization, and resilience [41]. Our study also found that the experience of depressive symptoms was significantly higher in the group with low resilience positivity.

Lee et al. found that nurses experience more depression, anxiety, and stress compared to other staff groups in hospitals [33]. However, our study found that depressive symptoms and occupation differ insignificantly. The data for this study in Korea was collected during the Omicron pandemic (138,993 new confirmed cases as of 00:00 on March 1). At the time, the number of beds dedicated to intensive care for COVID-19 increased six-fold as of the 1st of the last month to 1324 beds. Furthermore, in the last week of February, the average number of PCR tests exceeded 750,000 per day, exceeding the maximum test capacity of 850,000, as suggested by quarantine authorities. Owing to the rapid increase in new confirmed cases and frequent changes in quarantine guidelines, the burden of work in other occupations may be comparable to that of nurses. This may have resulted in an insignificant difference between depressive symptoms and hospital personnel. Therefore, rather than a simple comparison between general characteristics and each variable, comprehensive approaches should be considered based on the medical environment, working environment, and spread of the disease.

In the current situation, where there are concerns about the repeated spread of new infectious diseases even after the COVID-19 pandemic, most research on the effects of infectious disease outbreaks on mental health has targeted medical staff in private hospitals. There has been no research on personnel in military hospitals. Within the military, there is a peculiarity in the communal setting, which is vulnerable to the spread of infectious diseases. The evaluation of depressive symptoms of various personnel in military hospitals can provide important basic data for preparing military hospital personnel management plans. This can prepare for a situation where there is concern about the repeated spread of infectious diseases. In addition to the demographic and occupational characteristics investigated in this study, there is need for further research to consider factors such as military characteristics, family member types, regional spread of infectious diseases, changes in quarantine rules, duration of COVID-19 pandemic, religion, and corresponding monetary compensation. Additionally, to overcome depressive symptoms among hospital personnel, it is necessary to seek ways to improve individual resilience, especially positivity.

In the current study, resilience is important factor for odds of depressive symptoms in facing stress full status such as COVID-19. The resilience is one of the most important part of emotional intelligence (EI), and the military organization needed workers for high quality of EI to facing unpredictable stressful event [42]. Well-designed study from Spain highlighted that EI are closely related to teamwork, communication and job attitudes with job satisfaction [42]. Those factors also closely related to psychological impact in stress full situation [43]. Hence, to prepare such stressful event and related psychological problem of respondents, special training regarding EI and resilience were needed.

This study has several limitations. First, because of its cross-sectional design, the causality between depressive symptoms and resilience was not identified. Second, our sample may not be representative of military hospitals in the country as only one among four military hospitals for infectious diseases was analyzed. However, we tried to enhance the representativeness by setting the conditions as a hospital that has served as a hospital for infectious diseases within the last month and has a mission performance period of at least one year. Third, there were uncontrolled variables in this study owing to military specificity such as, frequent personnel changes (personnel changes every one to two years), the level of mission performance of military hospitals against infectious diseases (transition of all hospital functions, some functions, etc.), and duty alterations (medical staff were assigned duties regardless of their original work department). The current study did not show job-specific characteristics in the prevalence of depressive symptoms. The current study might have a relatively small sample size. More studies with larger sample sizes and comprehensive information of job-specific characteristics are needed to make clear strategies to prevent occupational depressive symptoms. 

Nevertheless, this study is significant in that it is the first study to examine the relationship between depressive symptoms and resilience among a variety of personnel in a military hospital that served as an infectious disease hospital during COVID-19. This study might provide useful basic data for human resource management in hospitals.

## 5. Conclusions

This study highlights the inverse relationship between resilience and depressive symptoms among military hospital staff. The low-resilience group show almost two times higher prevalence of depressive symptoms. Particularly, sub-categories of resilience in positivity patterns were found to be significantly associated with none of the depressive symptoms, even after controlling for demographic and occupational characteristics. To prevent depressive symptoms in such unfamiliar COVID-19 situations, the resilience of hospital staff is an important occupational health issue worldwide.

## Figures and Tables

**Table 1:** Demographic and occupational characteristics and Resilience according to depressive symptom.

Variable		Depressive Symptom						
		Depressive Symptom		Non-Depressive Symptom		Total		*p*-Value
		N	%	N	%	N	%	
Study participants		16	8.8	165	91.2	181	100.0	
Gender	Female	12	10.0	108	90.0	120	66.3	0.441
	Male	4	6.6	57	93.4	61	33.7	
Age(year)	20~29	5	13.9	31	86.1	36	19.9	0.294
	30~39	8	10.1	71	89.9	79	43.6	
	≥40	3	4.5	63	95.5	66	36.5	
Marital status	Married	8	9.0	81	91.0	89	49.2	0.945
	Single	8	8.7	84	91.3	92	50.8	
Occupation	Doctor	1	4.2	23	95.8	24	13.3	0.155
	Nurse	6	8.5	65	91.6	71	39.2	
	Nurse’s aide	2	4.3	45	95.7	47	26.0	
	Medical engineer	4	22.2	14	77.8	18	9.9	
	Administrative staff	3	14.3	18	85.7	21	11.6	
Medical experience for suspected or confirmed COVID-19	Yes	11	8.3	122	91.7	133	73.5	0.653
	No	5	10.4	43	89.6	48	26.5	
Work department (N = 133)	Isolation unit	7	6.6	99	93.4	106	79.7	0.167
	Screening clinic	4	14.8	23	85.2	27	20.3	
Total clinical career(year)	<1	1	3.5	28	96.6	29	16.0	0.116
	1~3	3	13.6	19	86.4	22	12.2	
	4~6	5	8.3	55	91.7	60	33.1	
	7~9	1	2.6	37	97.4	38	21.0	
	≥10	6	18.8	26	81.3	32	17.7	
Past medical support experience	Yes	8	14.0	49	86.0	57	31.5	0.095
	No	8	6.5	116	93.6	124	68.5	
Resilience (Total)	Low	14	20.3	55	79.7	69	38.1	<0.001
	High	2	1.8	110	98.2	112	61.9	
Self-Regulation	Low	11	19.0	47	81.0	58	32.0	0.001
	High	5	4.1	118	95.9	123	68.0	
Connections	Low	11	13.3	72	86.8	83	45.9	0.054
	High	5	5.1	93	94.9	98	54.1	
Positivity	Low	13	18.1	59	81.9	72	39.8	<0.001
	High	3	2.8	106	97.3	109	60.2	

**Table 2:** Demographic and occupational characteristics according to Resilience.

Variable		Resilience						
		Low-Resilience		High-Resilience		Total		*p*-Value
		N	%	N	%	N	%	
Study participants		69	38.1	112	61.9	181	100.0	
Gender	Female	46	38.3	74	61.7	120	66.3	0.934
	Male	23	37.7	38	62.3	61	33.7	
Age(year)	20~29	16	44.4	20	55.6	36	19.9	0.641
	30~39	28	35.4	51	64.6	79	43.6	
	≥40	25	37.9	41	62.1	66	36.5	
Marital status	Married	40	44.9	49	55.1	89	49.2	0.063
	Single	29	31.5	63	68.5	92	50.8	
Occupation	Doctor	8	33.3	16	66.7	24	13.3	0.211
	Nurse	27	38	44	62	71	39.2	
	Nurse’s aide	14	29.8	33	70.2	47	26	
	Medical engineer	11	61.1	7	38.9	18	9.9	
	Administrative staff	9	42.9	12	57.1	21	11.6	
Medical experience for suspected or confirmed COVID-19	Yes	47	35.3	86	64.7	133	73.5	0.199
	No	22	45.8	26	54.2	48	26.5	
Work department (N = 133)	Isolation unit	35	33	71	67	106	79.7	0.268
	Screening clinic	12	44.4	15	55.6	27	20.3	
Total clinical career(year)	<1	9	31	20	69	29	16	0.274
	1~3	8	36.4	14	63.6	22	12.2	
	4~6	27	45	33	55	60	33.1	
	7~9	10	26.3	28	73.7	38	21	
	≥10	15	46.9	17	53.1	32	17.7	
Past medical support experience	Yes	22	38.6	35	61.4	57	31.5	0.929
	No	47	37.9	77	62.1	124	68.5	

**Table 3:** Association of resilience and depressive symptom.

Variables		Odds Ratio (95% Confidential Interval)		
		Depressive Symptom		
		Model 1	Model 2	Model 3
Resilience	High	1.00 (reference)	1.00 (reference)	1.00 (reference)
	Low	2.00 (1.45–2.76)	8.87 (3.30–23.84)	10.30 (1.74–61.01)
Self-Regulation	High	1.00 (reference)	1.00 (reference)	1.00 (reference)
	Low	2.51 (1.79–3.52)	2.33 (1.06–5.11)	2.45 (0.55–10.87)
Connection	High	1.00 (reference)	1.00 (reference)	1.00 (reference)
	Low	1.29 (0.95–1.76)	1.56 (0.74–3.29)	1.69 (0.38–7.39)
Positivity	High	1.00 (reference)	1.00 (reference)	1.00 (reference)
	Low	1.80 (1.31–2.47)	2.45 (1.14–5.28)	13.90 (1.93–100.02)

Model 1: unadjusted; Model 2: adjusted by gender, age, marital status; Model 3: adjusted by gender, age, marital status, occupation, medical experience for suspected or confirmed COVID-19, total clinical career, past medical support experience.

## Data Availability

The datasets generated and/or analyzed during the current study are available from the corresponding author on reasonable request.

## Supplementary Materials

The following supporting information can be downloaded at: https://www.mdpi.com/article/10.3390/ijerph191811576/s1.
