# Supplementary figures and images for: Resilience and Depressive Symptoms among Medical Staff in a Military Hospital Dedicated to the Treatment of COVID-19
Source: Int J Environ Res Public Health. 2022 Sep 14;19(18):11576. doi: 10.3390/ijerph191811576 (PMC9517336; doi:10.3390/ijerph191811576)

**Figure S1. Flow chart of participants selection process**

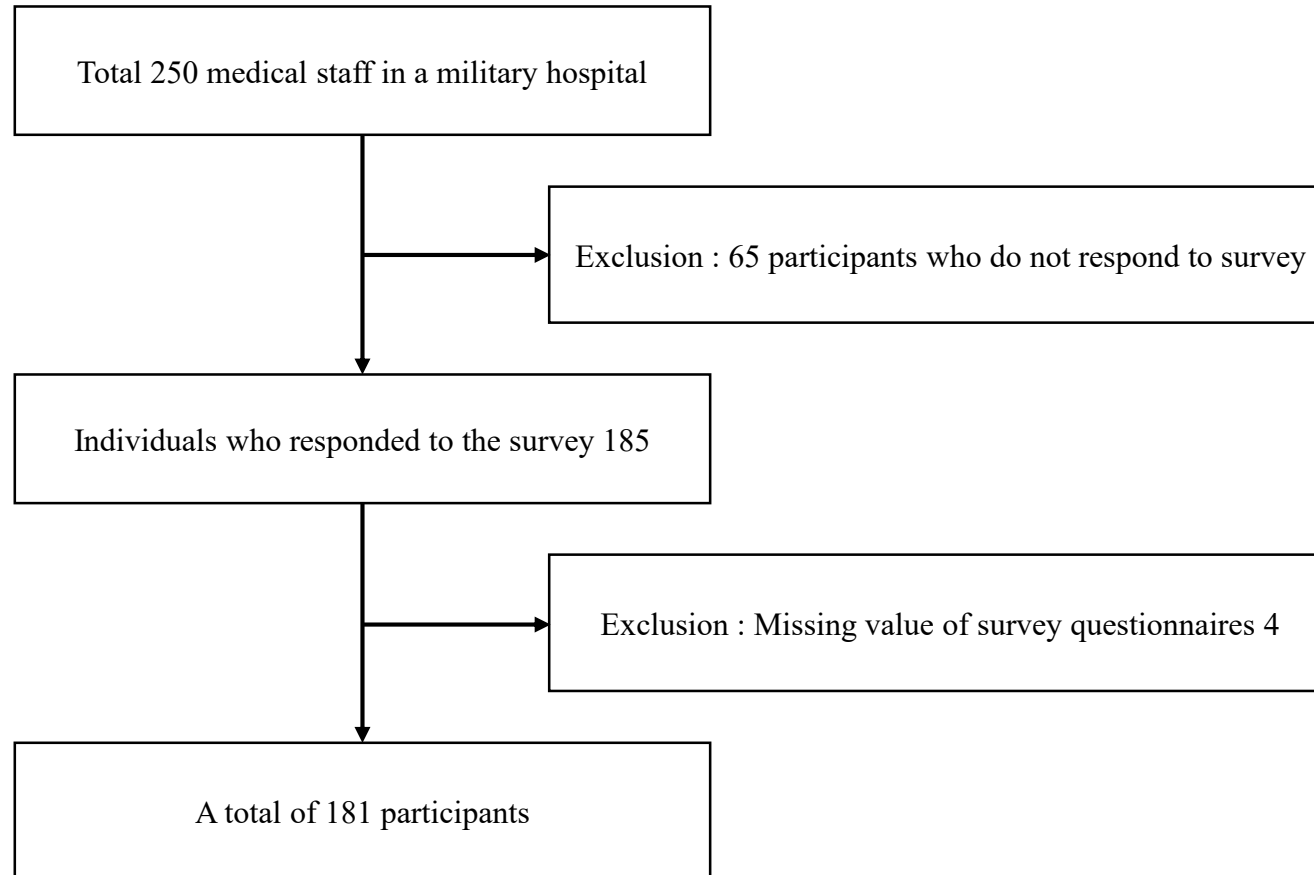

Supplement: Supplementary file 1 [file ijerph-19-11576-s001.zip › Figure S1.pdf]
